# Sephin1 Protects Neurons against Excitotoxicity Independently of the Integrated Stress Response

**DOI:** 10.3390/ijms21176088

**Published:** 2020-08-24

**Authors:** Asier Ruiz, Jone Zuazo, Carolina Ortiz-Sanz, Celia Luchena, Carlos Matute, Elena Alberdi

**Affiliations:** Departamento de Neurociencias, Universidad del País Vasco (UPV/EHU), Achucarro Basque Center for Neuroscience and Centro de Investigación Biomédica en Red de Enfermedades Neurodegenerativas (CIBERNED), 48940 Leioa, Spain; jzuazoibarra@gmail.com (J.Z.); carolinaortizsanz@gmail.com (C.O.-S.); cluchena001@ikasle.ehu.eus (C.L.); carlos.matute@ehu.eus (C.M.); elena.alberdi@ehu.eus (E.A.)

**Keywords:** excitotoxicity, integrated stress response, calpain, Sephin1, guanabenz, calcium, NMDA

## Abstract

Sephin1 is a derivative of guanabenz that inhibits the dephosphorylation of the eukaryotic initiation factor 2 alpha (eIF2α) and therefore may enhance the integrated stress response (ISR), an adaptive mechanism against different cellular stresses, such as accumulation of misfolded proteins. Unlike guanabenz, Sephin1 provides neuroprotection without adverse effects on the α2-adrenergic system and therefore it is considered a promising pharmacological therapeutic tool. Here, we have studied the effects of Sephin1 on N-methyl-D-aspartic acid (NMDA) receptor signaling which may modulate the ISR and contribute to excitotoxic neuronal loss in several neurodegenerative conditions. Time-course analysis of peIF2α levels after NMDA receptor overactivation showed a delayed dephosphorylation that occurred in the absence of activating transcription factor 4 (ATF4) and therefore independently of the ISR, in contrast to that observed during endoplasmic reticulum (ER) stress induced by tunicamycin and thapsigargin. Similar to guanabenz, Sephin1 completely blocked NMDA-induced neuronal death and was ineffective against AMPA-induced excitotoxicity, whereas it did not protect from experimental ER stress. Interestingly, both guanabenz and Sephin1 partially but significantly reduced NMDA-induced cytosolic Ca^2+^ increase, leading to a complete inhibition of subsequent calpain activation. We conclude that Sephin1 and guanabenz share common strong anti-excitotoxic properties with therapeutic potential unrelated to the ISR.

## 1. Introduction

Defective proteostasis is a major hallmark of several neurodegenerative diseases including Alzheimer’s disease, Parkinson´s disease, amyotrophic lateral sclerosis (ALS), frontotemporal dementia, and prion diseases [1]. Upon protein misfolding, the endoplasmic reticulum (ER) stress sensor protein kinase RNA-like ER kinase (PERK) phosphorylates the alpha subunit of the eukaryotic initiation factor 2 (eIF2α) and triggers a cytoprotective pathway called the integrated stress response (ISR) [2]. Phosphorylation of eIF2α at Ser51 (peIF2α) inhibits global translation to reduce the protein load but at the same time induces the translation of several ISR-specific genes such as chaperones and activating transcription factor 4 (ATF4) to restore proteostasis [3]. ATF4 is the main effector of the ISR and controls the expression of genes involved in amino acid metabolism and resistance to oxidative stress, among others [4]. Paradoxically, ATF4 is required as well to restore protein synthesis during later stages of the ISR [5]. ISR-mediated translational inhibition is terminated by peIF2α dephosphorylation, which is mediated by protein phosphatase 1 (PP1) upon binding to either regulatory subunit CReP or growth arrest and DNA damage-inducible protein 34 (GADD34). CReP is constitutively expressed and promotes peIF2α dephosphorylation in physiological conditions [6], whereas GADD34 reduces the ISR during ER stress in an ATF4-dependent manner following a negative feedback loop [7,8]. Interestingly, enhancing the ISR through pharmacological inhibition of GADD34-mediated peIF2α dephosphorylation provides neuroprotection in several models of neurodegeneration [9]. Salubrinal, pharmacological inhibitor of both CReP and GADD34 [10], provides neuroprotection [11] but it may have detrimental effects due to persistent translational inhibition. In contrast, GADD34 is expressed during stress and therefore its inhibition could have higher potential in a disease context [12]. The hypotensive drug guanabenz [13] enhances the ISR by inhibiting GADD34 [14] and protects neurons and oligodendrocytes in models of Parkinson´s disease and multiple sclerosis, respectively [15,16]. The therapeutic potential of guanabenz is limited as well due to its side effects on the α2-adrenergic system but led to the discovery and synthesis of its derivative Sephin1, a GADD34 specific inhibitor devoid of hypotensive activity [17]. Sephin1 ameliorates mouse models of Charcot–Marie–Tooth 1B and ALS [17], multiple sclerosis [18], and prion disease [19] and thus it has become a promising clinical strategy against protein misfolding diseases [1].

The overactivation of glutamate ionotropic receptors (NMDARs, AMPARs and KARs) is also involved in the pathogenesis of both neurodegenerative diseases [20] and brain ischemia [21]. An excessive Ca^2+^ overload into the cytosol, mainly through NMDARs, triggers the activation of calpains, oxidative stress and release of pro-apoptotic proteins, and eventually excitotoxic death of neurons [22]. In addition, there is some evidence linking excitotoxic conditions to ISR. Neurons exposed to kainate [23] and NMDA [24,25] showed a phosphorylation of eIF2α and in both cases excitotoxicity was attenuated by salubrinal, which enhances both constitutive (GADD34-independent) and stress-induced (GADD34-mediated) ISR. Importantly, Milhaud et al. [26] reported that guanabenz and other imidazolines inhibit NMDARs in a non-competitive manner and exert neuroprotection against excitotoxicity. However, the effects of Sephin1 on NMDA-induced neuronal excitotoxicity remain unknown. In this study, we show that the transient ISR induced by NMDA is followed by an ATF4-independent peIF2α dephosphorylation. However, Sephin1 strongly attenuates NMDA-induced excitotoxicity whereas it is ineffective against ER stress-produced neuronal death. We found that, similarly to guanabenz, Sephin1 reduces NMDA-triggered cytosolic Ca^2+^ load and calpain activity and provides neuroprotection against excitotoxicity in an ISR-independent manner.

## 2. Results

### 2.1. NMDA Induces an ISR-Independent peIF2α Dephosphorylation

**Figure 1:**
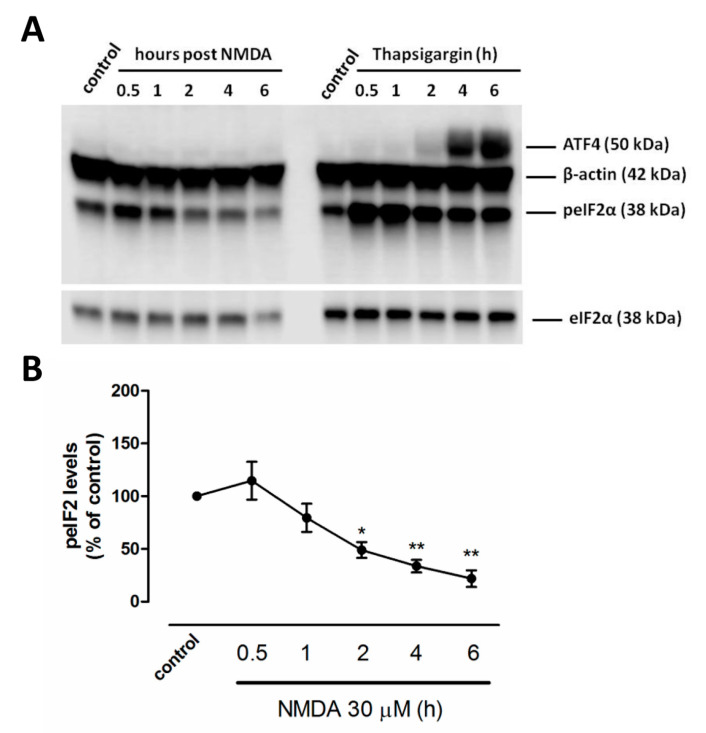
NMDAR activation induces activating transcription factor 4 (ATF4)-independent phosphorylation of eIF2α at Ser51 (peIF2α) dephosphorylation. (**A**) Cells were stimulated with NMDA (30 µM, 30 min) or thapsigargin (1 µM, always present) and harvested at indicated time points for immunoblotting of peIF2α, total eukaryotic initiation factor 2 (eIF2α), ATF4 and β-actin. (**B**) Quantification of peIF2α levels at indicated time points after NMDA treatment. Data represent normalized means ± SEM of three independent experiments. * *p* < 0.05; ** *p* < 0.01 compared with untreated cells (control), one-way ANOVA followed by Bonferroni´s post hoc test.

We previously reported that overactivation of NMDARs induces the ISR in cortical neurons [24,25] but its downstream effects are not fully understood. We analyzed the time course of eIF2α phosphorylation and ATF4 expression after NMDAR stimulation in cultured neurons. After a transient ISR, levels of peIF2α were strongly reduced to 49.05 ± 7.5%, 33.7 ± 5.9% and 21.9 ± 7.8% (of basal levels, 100%) after 2, 4 and 6 h respectively in NMDA-treated neurons (*n* = 3) (Figure 1A,B). Interestingly, NMDA-triggered ISR was not followed by ATF4 expression, in contrast to that induced by thapsigargin, a classical ER stressor that strongly activates the PERK-peIF2α-ATF4 pathway (Figure 1A). Thus, results indicate that peIF2α dephosphorylation after NMDAR activation is independent of the peIF2α/ATF4 pathway.

### 2.2. Sephin1 Strongly Attenuates NMDA-Induced Excitotoxicity But Not ER Stress-Induced Neuronal Death

**Figure 2:**
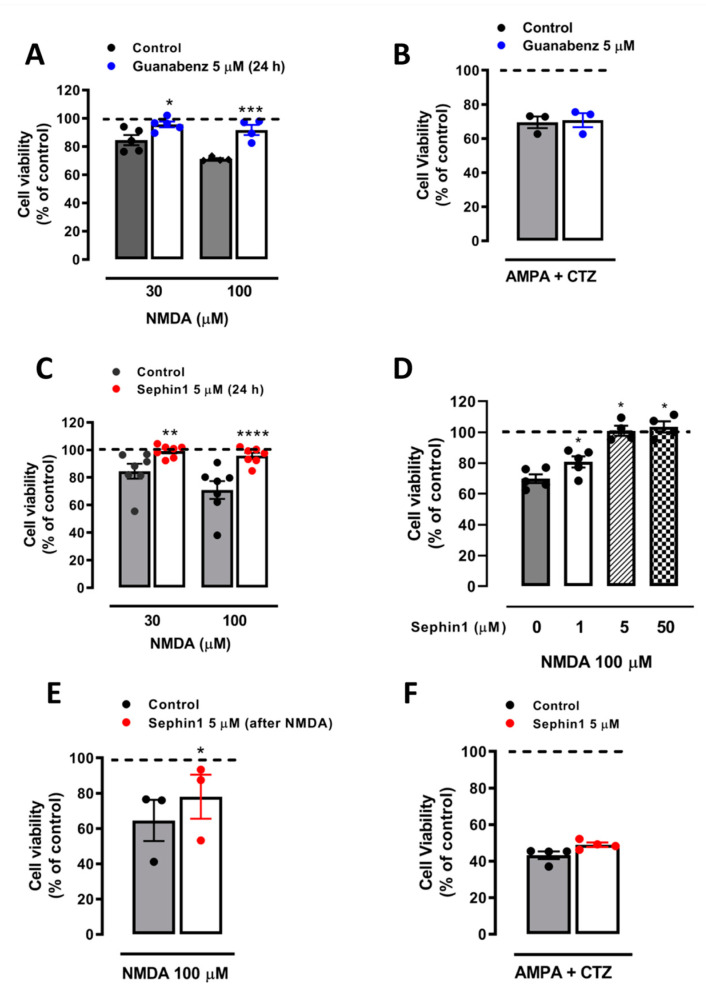
Sephin1 and guanabenz strongly attenuate NMDA-induced (but not AMPA/CTZ-induced) excitotoxicity. (**A**) Neurons were stimulated with NMDA (30 and 100 µM, 30 min) in the presence or absence of guanabenz (5 µM, present before, during, and after NMDA addition). * *p* < 0.05, *** *p* < 0.001 compared with control (NMDA alone), one-way ANOVA followed by Bonferroni´s post hoc test. (**B**) Neurons were treated with AMPA (25 µM, 30 min) and cyclothiazide (CTZ, 100 µM) in the presence or absence of guanabenz (5 µM, present before, during, and after AMPA/CTZ addition) and paired Student’s t-test. (**C**) Neurons were stimulated with NMDA (30 and 100 µM, 30 min) in the presence or absence of Sephin1 (5 µM, present before, during, and after NMDA addition). ** *p* < 0.01, **** *p* < 0.0001 compared with control (NMDA alone), one-way ANOVA followed by Bonferroni´s post hoc test. (**D**) Neurons were stimulated with 100 µM of NMDA for 30 min in the presence or absence of increasing concentrations of Sephin1 (1, 5, and 50 µM, added 1 h before NMDA addition and washed after NMDA). * *p* < 0.05 compared with control (NMDA alone), one-way ANOVA followed by Bonferroni´s post hoc test. (**E**) Neurons were stimulated with 100 µM of NMDA for 30 min in the presence or absence of Sephin1 (5 µM, added after NMDA wash). * *p* < 0.05 compared with control (NMDA alone), paired Student’s t-test. (**F**) Cells were treated with 25 µM of AMPA plus CTZ in the presence or absence of Sephin1 (5 µM, added 1 h before AMPA/CTZ addition), paired Student’s t-test. In all cases, cell viability was assessed 24 h later by the quantification of vital dye calcein-AM fluorescence. Data represent means ± SEM of normalized calcein fluorescence values.

Guanabenz inhibits NMDARs and protects cerebellar and striatal neurons from excitotoxicity [26]. Similarly, we observed that 5 µM of guanabenz (present 1 h before, during, and after NMDA) increased viability of cortical neurons exposed to 30 and 100 µM of NMDA from 84.5 ± 3.6% to 95.6 ± 2% (*n* = 5) and from 71.1 ± 0.7% to 91.7 ± 3.6% (*n* = 4) of control (untreated cells, 100% viability), respectively (Figure 2A). However, guanabenz was ineffective against AMPAR-mediated excitotoxicity, induced by the addition of AMPA (25 µM) plus 100 µM of cyclothiazide (CTZ), an inhibitor of AMPAR desensitization (Figure 2B). Thus, guanabenz showed selectivity against excitotoxicity mediated by NMDA-type glutamate ionotropic receptors. Next, we tested the ability of Sephin1, a derivative of guanabenz, to attenuate NMDA-induced excitotoxicity in cortical neurons. Like guanabenz, the presence of Sephin1 before, during, and after NMDA treatment increased viability of neurons from 69.4 ± 7.4% to 96.1 ± 2.6% (*n* = 6) of control (untreated cells, 100% viability) (Figure 2C). Furthermore, Sephin1 applied only before and during NMDA treatment also completely inhibited excitotoxicity at low micromolar concentration. Specifically, Sephin1 protected neurons against NMDA-induced toxicity in a dose-dependent manner from 68.4 ± 3% to 78.9 ± 4%, 100.9 ± 3.2% and 103.4 ± 3.6% viability (*n* = 5) at 1, 5 and 50 µM of Sephin1 (Figure 2D). Additionally, when Sephin1 was added overnight immediately after NMDA wash, it partially increased viability from 64.5 ± 11.2% to 78 ± 24.1% (*n* = 3) of control (untreated cells, 100%) (Figure 2E). Like guanabenz, Sephin1 did not provide neuroprotection against toxic stimuli induced by non-desensitizing AMPA receptor activation (Figure 2F).

**Figure 3:**
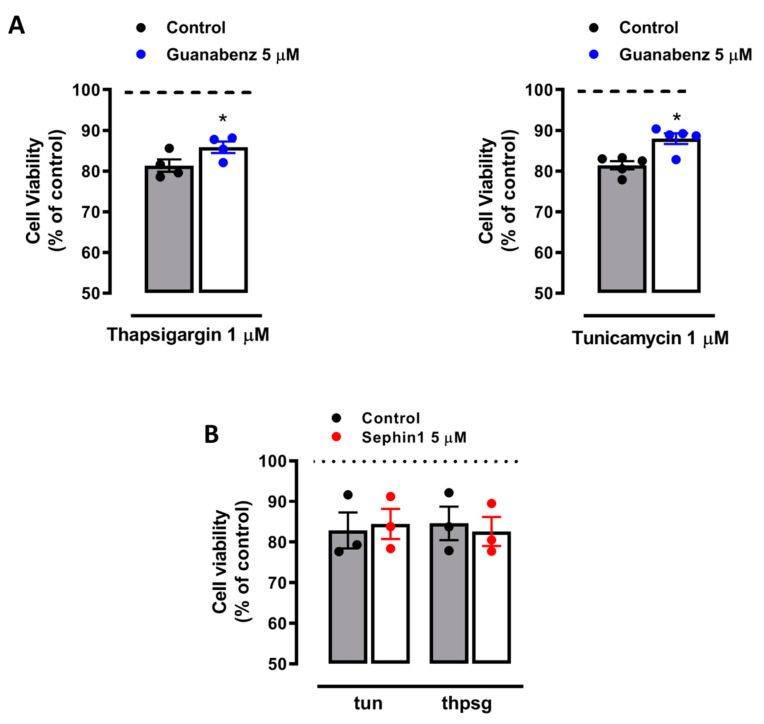
Guanabenz but not Sephin1 reduces ER stress-induced neuronal death. Primary cultured neurons were exposed to tunicamycin and thapsigargin (1 µM both, 24 h) in the absence or presence of (**A**) guanabenz (5 µM, 24 h) and (**B**) Sephin1 (5 µM, 24 h). The number of viable cells was assessed by fluorescent vital dye calcein-AM 24 h later. Histograms represent means ± SEM of calcein fluorescence values normalized to controls. * *p* < 0.05 compared with cells treated with tunicamycin or thapsigargin alone, paired Student’s t-test.

On the other hand, both guanabenz and Sephin1 protect against ER stress-induced cell death by ISR enhancement in models of proteostasis disruption [14,17]. In cortical neurons exposed to classical ER stressors such as thapsigargin and tunicamycin, guanabenz partially rescued neuronal viability from 81.3 ± 1.5% to 85.8 ± 1.4% (*n* = 4) and from 81.4 ± 1% to 87.9 ± 1.3% (*n* = 5), respectively (Figure 3A). However, Sephin1 did not protect against either thapsigargin or tunicamycin insults (*n* = 3) (Figure 3B). These results indicate that Sephin1 modulates early molecular events triggered by NMDAR signaling, and prevents excitotoxic death independently of the ISR.

### 2.3. Sephin1 and Guanabenz Reduce NMDA-Induced Cytosolic Ca^2+^ Overload

**Figure 4:**
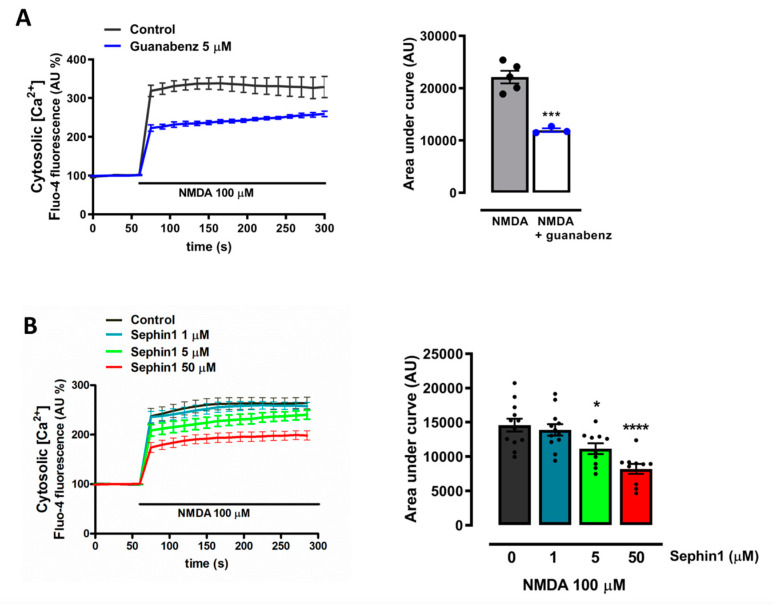
Sephin1 and guanabenz reduce NMDA-induced cytosolic Ca^2+^ load. (**A**) Fluo-4-loaded neurons were exposed to NMDA (100 µM) in the absence or presence of guanabenz (5 µM) and cytosolic Ca^2+^ load quantified. *** *p* < 0.001 compared to control cells (NMDA alone), unpaired Student’s t-test. (**B**) Neurons were treated in the absence or presence of Sephin1 (1, 5 and 50 µM) and cytosolic Ca^2+^ load quantified. * *p* < 0.05, **** *p* < 0.0001 compared with control (NMDA alone), one-way ANOVA followed by Bonferroni´s post hoc test. Traces show normalized means ± SEM of several fields of view (*n*) from no less than three independent cultures. Statistical significance was calculated from normalized average ± SEM of the area under the curve.

Since excitotoxic cell death is mainly triggered by an intracellular Ca^2+^ homeostasis disruption, we next analyzed the effects of both guanabenz and Sephin1 on NMDA-induced [Ca^2+^]_cyt_ overload. As expected, guanabenz reduced the [Ca^2+^]_cyt_ increase triggered by NMDA from a peak amplitude of 338.4 ± 16.5% (*n* = 5) to 259.5 ± 7% (*n* = 3) compared with resting levels (100%) (Figure 4A). Correspondingly, excitotoxic [Ca^2+^]_cyt_ rise was attenuated in a dose-dependent manner by 5 and 50 µM of Sephin1 from 264.1 ± 11.6% (*n* = 12) to 240.4 ± 8.9% (*n* = 10) and 199.3 ± 8.9% (*n* = 10), respectively, whereas 1 µM of Sephin1 caused no detectable changes (Figure 4B).

### 2.4. Sephin1 and Guanabenz Block NMDA-Induced Calpain Activation

**Figure 5:**
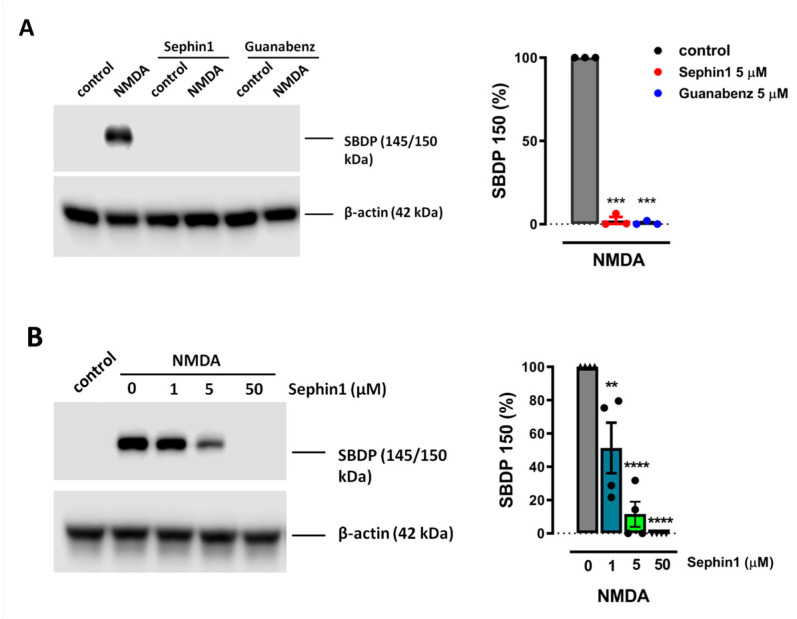
Sephin1 and guanabenz inhibit NMDA-induced calpain activation. (**A**) Neurons were exposed to NMDA (100 µM, 30 min) in the absence or presence of Sephin1 or guanabenz (5 µM) and harvested 4 h later for the detection of αII-spectrin breakdown products (SBDP) and β-actin by Western blot. *** *p* < 0.001 compared with cells treated with NMDA alone (100%), paired Student’s t-test. (**B**) Neurons were treated with NMDA (100 µM, 30 min) in the absence or presence of increasing concentrations of Sephin1 (1, 5, 50 µM) and harvested as above. ** *p* < 0.05, **** *p* < 0.0001 compared with control (NMDA alone), one-way ANOVA followed by Bonferroni´s post hoc test. In all cases, for the quantification of calpain activity, SBDP150/145 signal was measured and normalized to β-actin values.

We previously showed that blockade of calpain activity correlates with neuroprotection in the excitotoxic paradigm used in the current study [25,27]. Therefore, we next studied whether guanabenz and Sephin1 in particular modulated the cleavage of αII-spectrin into 145/150 breakdown products (SBDP 145/150), which is indicative for calpain activity [28]. Pre-incubation of neurons with guanabenz or Sephin1 at 5 µM completely inhibited the production of SBDP 145/150 to 2.2 ± 2% (*n* = 3) and 0.7 ± 0.7% (*n* = 3) of cells treated with NMDA alone (100%) (Figure 5A). To further analyze the effects of Sephin1 on NMDA-induced calpain activation, we quantified αII-spectrin cleavage in the presence of increasing concentrations of the drug previously used in toxicity and Ca^2+^ imaging experiments. SBDP 145/150 were reduced in a dose-dependent manner to 51.3 ± 15.1% (*n* = 4), 11.5 ± 7.5% (*n* = 4), and 0.01 ± 0.01% (*n* = 4) of control (100%) by 1, 5 and 50 µM of Sephin1, respectively (Figure 5B). These results suggest that inhibition of NMDARs and subsequent Ca^2+^ overload attenuation by guanabenz and Sephin1 results in calpain inhibition-dependent neuroprotection.

## 3. Discussion

A cytoprotective ISR is triggered by a variety of stresses such as protein misfolding and aggregation, oxidative stress, and mitochondrial dysfunction and its pharmacological enhancement provides neuroprotection in several models of neurodegeneration [29]. On the other hand, NMDAR-mediated excitotoxicity is involved as well in several brain diseases [20] but its link with the ISR is not fully understood. We previously showed that in cortical neurons NMDA induces an ISR that correlates as well with neuroprotection [24,25]. Thus, we hypothesized that increasing eIF2α phosphorylation by the ISR enhancer Sephin1 attenuates the excitotoxicity produced by NMDAR overactivation. NMDAR activation induced a transient ISR that was followed by a strong peIF2α dephosphorylation below basal levels. In contrast to the ISR induced by the ER stressor thapsigargin, NMDA-triggered ISR did not result in ATF4 expression. Since GADD34 is expressed downstream ATF4 during stress [5], these results indicate that peIF2α dephosphorylation after NMDA was independent of GADD34. Strikingly, both guanabenz and Sephin1, that protect against proteostasis disruption and ER stress by the inhibition of GADD34-mediated peIF2α dephosphorylation [14,17], strongly reduced NMDA-induced neuronal death. Indeed, at concentrations previously used to enhance the ISR and prevent ER stress-induced death in HeLa cells [14,17] guanabenz, and Sephin1 in particular, completely blocked excitotoxicity even when they were washed immediately after NMDA stimuli. Interestingly, addition of Sephin1 after NMDA stimuli still increased partially the viability of neurons. This observation further supported the notion that Sephin1 was mainly modulating early NMDAR signaling and not a delayed downstream event, such as GADD34 activity. Furthermore, whereas guanabenz modestly reduced neuronal death produced by classical ER stressors, Sephin1 was totally ineffective. Therefore, we conclude that the mechanism of the robust neuroprotection against excitotoxicity provided by guanabenz and Sephin1 in our experimental paradigm is independent of the ISR. These data are in agreement with a previous report showing that guanabenz and other imidazolines block NMDARs. Thus, guanabenz inhibits NMDA-induced currents in a reversible and non-competitive manner, which correlates with robust neuroprotection in cerebellar and striatal neurons [26]. Consistent with these findings, we found that in cortical neurons, both guanabenz and its derivative Sephin1 protected cortical neurons selectively against NMDA-induced excitotoxicity because of the attenuation of cytosolic Ca^2+^ overload and subsequent calpain activation. We previously reported that NMDA-induced toxicity in cortical neurons correlates with activation of calpains [25,27], which are Ca^2+^-activated proteases that play a critical role in ischemic and excitotoxic neuronal damage [30,31]. In conditions of complete neuroprotection, guanabenz and Sephin1 totally reduced the cleavage of αII-spectrin, indicative of calpain activity [28]. We excluded the possibility that the inhibitors could directly inhibit calpains, since neither guanabenz nor Sephin1 protected against AMPA-induced excitotoxicity, which robustly activates calpains [27]. Further analysis of Sephin1 blockade of αII-spectrin cleavage by Sephin1 showed a dose-dependent inhibition of calpain activation, which correlated with those obtained in neuroprotection and [Ca^2+^]_cyt_ experiments. Interestingly, we observed that low micromolar concentration of Sephin1 only moderately reduced NMDA-triggered cytosolic Ca^2+^ load but exerted a huge impact on both calpain activity and toxicity, suggesting a “threshold effect”. Indeed, doses as low as 1 µM of Sephin1 did not produce detectable NMDA-mediated [Ca^2+^]_cyt_ changes, yet attenuated calpain activity and toxicity.

Although their inhibitory effects on GADD34 have been questioned [32], guanabenz and Sephin1 were further confirmed as GADD34 inhibitors by another high-profile study [33]. Devoid of αII-adrenergic system activity, Sephin1 is considered a well-tolerated ISR enhancer with potential for the treatment of neurodegenerative diseases involving proteostasis disruption [1]. However, in the current study we provide evidence that similarly to guanabenz, Sephin1 has strong anti-excitotoxic properties. To the best of our knowledge, we show for the first time that Sephin1 reduces NMDA-induced [Ca^2+^]_cyt_ overload and calpain activation, leading to a strong attenuation of neuronal excitotoxicity independently of the ISR. This feature provides Sephin1 with further therapeutic potential and should be taken into account in clinical studies.

## 4. Materials and Methods

### 4.1. Animals

Animal protocols were approved on 21 December 2017 by the Animals Ethics and Welfare Committee of the University of the Basque Country (Ethics approval number M20_2017_087) and performed in accordance with the Directives of the European Union on animal ethics and welfare. All efforts were made to minimize the number of animals used and their suffering.

### 4.2. Reagents

Neurobasal^®®^ medium, B-27 supplement, 100× antibiotic–antimycotic (10.000 units/mL penicillin, 10.000 µg/mL streptomicyn and 25 µg/mL Fungizone^®®^) and calcein-AM (calcein acetoxymethyl ester) were purchased from ThermoFisher Scientific (Waltham, MA, USA). NMDA (N-methyl-D-aspartic acid), Sephin1, guanabenz, HBSS, glycine, poly-L-ornithine, glutamine, thapsigargin and tunycamicin were obtained from MilliporeSigma/Merck (Burlington, MA, USA). AMPA (α-amino-3-hydroxy-5-methyl-4-isoxazolepropionic acid) and cyclothiazide (CTZ) were obtained from Tocris Biosciences (Bristol, UK).

### 4.3. Neuronal Primary Culture

Neurons were cultured from the cortical lobes of E18 embryos obtained from Sprague–Dawley rats according to previously described procedures [34,35]. Cells were resuspended in 10% FBS-containing Neurobasal^®®^ medium supplemented with B27, glutamine (2 mM) and antibiotic-antimycotic mixture, and seeded at 1.5 × 10^5^ cells per well onto poly-L-ornithine-coated 48 well plates. For live cell imaging experiments, cells were plated onto poly-L-ornithine-coated glass-bottom μ-dishes (Ibidi GmbH, Gräfelfing, Germany). The medium was replaced by serum-free, supplemented Neurobasal^®®^ medium 24 h after plating and maintained in a humidified CO_2_ incubator (5% CO_2,_ 37 °C). Cultured neurons were used between 8–10 days after plating.

### 4.4. Western Blotting

For protein extraction, triplicates of 1.5 × 10^5^ cells were washed with PBS, harvested and resuspended in 50 µl of ice-cold electrophoresis sample buffer. Lysates were boiled for 10 min, subjected to electrophoresis using Criterion™ TGX™ Precast gels and transferred to Trans-Blot^®®^ Turbo™ Midi PVDF Transfer Packs (Bio Rad, Hercules, CA, USA). Membranes were blocked with 5% skimmed powdered milk and 5% normal serum in TBS containing 0.05% Tween 20 (TBST) and incubated overnight at 4 °C with primary antibodies diluted in TBST containing 5% BSA: anti-ATF4 (Cell Signaling, Danvers, MA, USA), anti-αII Spectrin (1:1000; Santa Cruz Biotechnology, Dallas, TX, USA), anti-peIF2 and anti-eIF2 (1:1000; Cell Signaling), and anti-β actin (1:2000; MilliporeSigma, Burlington, MA, USA). Membranes were washed three times with TBST and incubated with horseradish peroxidase-conjugated secondary antibodies (1:2000, Sigma) in 5% skimmed milk, 1% normal serum in TTBS for 2 h at room temperature. After three washes, blots were developed using enhanced chemiluminiscence according to the manufacturer´s instructions (Super Signal West Dura, Pierce, Rockford, IL, USA) in a C-Digit^®®^ Blot Scanner (Li-Cor, Lincoln, NE, USA). Chemiluminiscence bands were quantified using Image Studio™ software (Li-Cor) and values were normalized to β-actin or to total eIF2α signal when indicated and provided as the mean ± SEM of at least three independent experiments.

### 4.5. Toxicity Assays

In excitotoxicity experiments, neurons were treated with NMDA in HBSS (free of Ca^2+^ and Mg^2+^) containing 2.6 mM CaCl_2_, 10 mM glucose, and 10 µM glycine or with 25 µM of AMPA plus 100 µM cyclothiazide in supplemented Neurobasal^®®^ for 30 min at 37 °C and washed. In ER stress-induced toxicity assays neurons were overnight exposed to 1 µM of thapsigargin or tunicamycin. In all cases, cell viability was estimated 24 h later by fluorescent vital dye calcein-AM by a Synergy™ H4 Hybrid microplate reader (BioTek, Winooski, VT, USA). All experiments were performed with four replicates and the values provided are the normalized mean ± SEM of at least three independent cultures.

### 4.6. Cytosolic Ca^2+^ Imaging

Time-lapse recordings of [Ca^2+^]_cyt_ were performed as previously described [25] with modifications. Neurons were incubated with cell-permeant Fluo-4 AM dye (1 µM; ThermoFisher Scientific) in Ca^2+^ and Mg^2+^-free HBSS containing 20 mM HEPES, 10 mM glucose, 10 µM glycine, and 2.6 mM CaCl_2_ for 30 min at 37 °C followed by 20 min wash to allow de-esterification. Images were obtained through a 63× objective by inverted LCS SP2 or TCS SP8X confocal microscopes (Leica, Wetzlar, Germany) at an acquisition rate of 1 frame/15 s during 5 min. For data analysis, a homogeneous population of 15–25 cells was selected in the field of view and neuronal somata selected as regions of interest (ROIs). Background corrected values were expressed as *F*/*F*_0_ ± SEM (%) in which *F* represents the fluorescence value for a given time point and *F*_0_ represents the mean of the resting fluorescence level.

### 4.7. Data Analysis

Data are given as mean ± SEM (*n*), where *n* refers to the number of cultures assayed, each obtained from a different group of animals. In live cell confocal imaging experiments, *n* refers to number of fields of view acquired, obtained from at least three independent cultures. In each field of view at least 15–25 neuronal somata were identified and recorded. For statistical analysis of the [Ca^2+^]_cyt,_ the basal line-extracted area under curve was calculated from single cell imaging time-lapse curves. Statistical significance was determined using Student’s t-test or one way analysis of variance (ANOVA) followed by Bonferroni´s post hoc test. At *p* < 0.05, mean difference was considered to be statistically significant.
